# Reperfusion Therapies for Acute Ischemic Stroke in COVID-19 Patients: A Nationwide Multi-Center Study

**DOI:** 10.3390/jcm11113004

**Published:** 2022-05-26

**Authors:** Justina Jurkevičienė, Mantas Vaišvilas, Rytis Masiliūnas, Vaidas Matijošaitis, Antanas Vaitkus, Dovilė Geštautaitė, Saulius Taroza, Paulius Puzinas, Erika Galvanauskaitė, Dalius Jatužis, Aleksandras Vilionskis

**Affiliations:** 1 Stroke Centre, Republican Vilnius University Hospital, 04130 Vilnius, Lithuania; mantas.vaisvilas@rvul.lt; 2 Center of Neurology, Vilnius University, 08661 Vilnius, Lithuania; rytis.masiliunas@santa.lt (R.M.); dalius.jatuzis@santa.lt (D.J.); 3 Department of Neurology, Lithuanian University of Health Sciences, 50009 Kaunas, Lithuania; vaidas.matijosaitis@kaunoklinikos.lt (V.M.); antanas.vaitkus@kaunoklinikos.lt (A.V.); dovile.gestautaite@kaunoklinikos.lt (D.G.); 4 Laboratory of Behavioral Medicine (Palanga), Neuroscience Institute, Lithuanian University of Health Sciences, 00135 Palanga, Lithuania; saulius.taroza@lsmuni.lt; 5 Department of Neurology, Republican Panevėžys Hospital, 35144 Panevėžys, Lithuania; pauliuspuzinas@gmail.com; 6 Department of Neurology, Republican Šiauliai Hospital, 76231 Šiauliai, Lithuania; erika.galvanauskaite@siauliuligonine.lt; 7 Clinic of Neurology and Neurosurgery, Vilnius University, 03101 Vilnius, Lithuania; aleksandras.vilionskis@rvul.lt

**Keywords:** COVID-19, ischemic stroke, thrombolysis, thrombectomy, Lithuania, reperfusion therapies, outcomes, safety

## Abstract

(1) Background: Acute ischemic stroke (AIS) is a possible complication of the coronavirus disease 2019 (COVID-19). Safety and efficacy data on reperfusion therapies (RT)—intravenous thrombolysis and endovascular treatment (EVT)—in stroke patients with COVID-19 is lacking. (2) Methods: We performed a retrospective nationwide multi-center pair-matched analysis of COVID-19 patients with AIS who underwent RT. We included adult COVID-19 patients with AIS who were treated with RT between 16 March 2020 and 30 June 2021. All subjects were paired with non-infected controls, matched for age, sex, stroke arterial vascular territory, and RT modality. The primary outcome measure was a favorable functional outcome defined by the modified Rankin scale (mRS 0–2). (3) Results: Thirty-one subjects and thirty-one matched controls were included. The median baseline National Institutes of Health Stroke Scale (NIHSS) score was higher in the COVID-19 group (16 vs. 12, *p* = 0.028). Rates of ischemic changes and symptomatic intracerebral hemorrhages did not differ significantly between the two groups at 24 h after RT. The median NIHSS 24 h after reperfusion remained significantly higher in the COVID-19 group (16 vs. 5, *p* = 0.003). MRS 0–2 at discharge was significantly less common in COVID-19 patients (22.6% vs. 51.8%, *p* = 0.018). Three-month mortality was 54.8% in the COVID-19 group versus 12.9% in controls (*p* = 0.001). (4) Conclusion: Reperfusion therapies on AIS in COVID-19 patients appear to be safe; however, functional outcomes are significantly worse, and 3-month mortality is higher.

## 1. Introduction

In December 2019, a cluster of patients with pneumonia caused by a novel severe acute respiratory coronavirus 2 (SARS-CoV-2) was first described in Wuhan, China [1]. Due to the vast spread of the virus across the globe, a pandemic was declared in March 2020. Ever since, a growing number of publications regarding extrapulmonary manifestations of coronavirus disease (COVID-19) arose. Neurologic manifestations of both the central and the peripheral nervous system described included COVID-19 encephalitis, acute disseminated encephalomyelitis, epileptic seizures, neuromuscular symptoms, acute demyelinating polyneuropathies, and their variants, as well as acute cerebrovascular syndromes [2,3,4,5,6,7,8]. It has been postulated that COVID-19 patients are at an increased risk for stroke, although the true causality is yet uncertain [9].

The first COVID-19 case in Lithuania was confirmed in late February 2020, followed shortly by the introduction of a strict nationwide lockdown. Despite thousands of daily new confirmed cases and the need for allocation of specific healthcare resources, emergency stroke services were operating in all major stroke centers across the country throughout the pandemic at full capacity [10,11]. Both intravenous thrombolysis (IVT) and endovascular treatment (EVT) were used continuously for acute ischemic stroke (AIS) in COVID-19 patients. However, data on the safety of reperfusion therapies (RT) in the COVID-19 population is scarce, and potential adverse effects of RTs could be life-threatening. Therefore, we sought to evaluate the safety and outcomes of reperfusion therapies in COVID-19 patients with AIS in a nationwide pair-matched retrospective study.

## 2. Materials and Methods

We conducted a multi-center retrospective pair-matched analysis of reperfusion therapy in COVID-19 patients with AIS across all six comprehensive stroke centers (CSCs) in Lithuania [12].

**Data collection.** The data were extracted retrospectively from electronic health records. We collected demographic data (age, gender), cardiovascular risk factors (hypertension, dyslipidemia, smoking, diabetes, atrial fibrillation, presence of symptomatic internal carotid artery (ICA) >70% or intracranial artery stenosis > 70% on computed tomography angiography), clinical (hypoxemia, body temperature, blood pressure on admission) and laboratory test data (white blood cell (WBC) and lymphocyte count, C reactive protein (CRP) and D-dimer levels on admission), head computed tomography (CT) findings (Alberta Stroke Programme Early CT Score (ASPECTS) on admission, ischemic changes on CT scan 24 h after RT), median timeliness metrics (onset-to-door (OTD), door-to-needle (DTN) and door-to-puncture (DTP) times), National Institute of Health Stroke Scale (NIHSS) on admission, at 24 h after reperfusion therapy, and on day 7 after stroke or at discharge (whichever occurred first) and reperfusion therapy data (treatment modality, Thrombolysis in Cerebral Infarction (TICI) score). Neurologic (symptomatic intracerebral hemorrhage (sICH), cerebral edema), COVID-19-related, and other complications (urinary tract infection, pulmonary embolism, myocardial infarction, acute heart failure, pulmonary edema, other organ dysfunction, or major bleeding) were collected. Patient functional outcomes corresponding to modified Rankin Scale (mRS) score at discharge, as well as in-hospital and 3-month mortality rates, were retrieved.

**Patient selection.** We included adult (18 years old or older) AIS patients with diagnosed acute COVID-19 infection prior to or on admission to a CSC, treated with reperfusion therapy (IVT, EVT, or both) between 16 March 2020 and 30 June 2021. Our patients had not received full vaccination doses. COVID-19 status was confirmed by a nasopharyngeal swab SARS-CoV-2 real-time polymerase chain reaction (RT-PCR). Patients who recovered from COVID-19 according to the epidemiological criteria at the time of index AIS were excluded from the analysis despite having a positive SARS-CoV2 RT-PCR test result.

**Control group.** Each patient from the subject group was weighted against a control. All control patients were treated in one of the 6 Lithuanian CSCs during the study period and were not concomitant with a COVID-19 infection. In addition, control subjects were matched for age (±5 years), gender, stroke arterial vascular territory, and type of reperfusion therapy (IVT, EVT, or both). To avoid selection bias, cases for this group were collected by independent stroke physicians, who were not part of this study, and were only informed about matching criteria.

**Outcomes.** The primary outcome measure was a favorable functional outcome, defined as the mRS score of 0–2 on the day of discharge.

Secondary outcome measures included: early neurological improvement, defined as reduction of NIHSS score by 4 points or more or score 0–1 at 24 h after reperfusion therapy; change in NIHSS score 24 h after reperfusion therapy; change in NIHSS score 7 days after stroke onset or on discharge (whichever occurred first); neurological complications of reperfusion therapy: sICH was classified using the Safe Implementation of Thrombolysis in Stroke-Monitoring Study (SITS-MOST) classification (parenchymal hemorrhage type 2, 22–36 h after treatment leading to neurologic deterioration 4 points or more on NIHSS from baseline or lowest NIHSS or leading to death as previously reported) [13], and cerebral edema; in-hospital mortality rate; mortality rate 3 months after stroke.

To investigate the effects of clinical and laboratory factors (evaluated on admission) on the likelihood of favorable functional outcome (mRS 0–2) on the day of discharge and of 3-month mortality after stroke and reperfusion therapies, multivariate logistic regression models were built.

**Statistical analysis.** Statistical analysis was performed using the IBM SPSS Statistics for Windows, Version 26 (IBM SPSS Statistics for Windows, IBM Corporation, Armonk, NY, USA). The Kolmogorov–Smirnov test was used to verify the normality of the distribution of continuous variables. The qualitative variables were expressed as absolute frequencies and percentages. For continuous data, the mean and standard deviation (SD) or median and interquartile range (IQR) were reported, as appropriate. The Student’s *t* test (for normally distributed data) or the Mann–Whitney U test (for not normally distributed data) was used for the continuous variables and the Chi-square test for the categorical variables. *p* < 0.05 was considered to be statistically significant. The significant predictors (using a significance level of <0.1) in the univariate analysis were included in the multivariate analysis, and the entered method was applied for the logistic regression model to determine the predictors for a favorable functional outcome (mRS 0–2) on discharge and 3-month mortality after stroke. The odds ratio (OR) and 95% confidence interval (95% CI) were calculated.

## 3. Results

### 3.1. Demographic, Clinical, and Stroke-Related Data

Thirty-one pairs of subjects and matched controls were included in the study. The mean age was 74.0 years in COVID-19-positive AIS patients and 73.7 years in controls. Forty females (64.5%) comprised the entire cohort. Prevalence of stroke risk factors did not differ statistically significantly between the two groups. Fourteen (22.5%) patients underwent IVT, thirty (48.4%) patients were treated with EVT, and eighteen (29.1%) patients received bridging therapy. Fifty-six (90.3%) patients in the entire cohort were diagnosed with anterior circulation stroke. The detailed demographic data and stroke risk factors are displayed in Table 1.

The median NIHSS score on admission was significantly higher in the COVID-19 patient group compared to controls (16 [10–19] vs. 12.5 (5–15), *p* = 0.028). The timeliness metrics (OTD, DTN, and DTP times) did not differ significantly between the two groups. Albeit not significant, the OTD time was longer for COVID-19 patients as compared to controls (126 (83–218) vs. 95 (66–205) minutes, respectively). The ASPECTS score on admission also did not differ significantly.

As expected, the baseline body temperature was statistically significantly higher in COVID-19 patients compared to controls (*p* = 0.025), while the rate of hypoxemia and arterial blood pressure on admission did not differ significantly (Table 2). A significantly lower lymphocyte count (*p* = 0.013) and higher CRP values (*p* < 0.001) were observed in the COVID-19 group compared to controls, while total WBC count and D-dimer concentration on admission did not differ.

### 3.2. Primary and Secondary Outcomes

Only 22.6% of COVID-19 patients with AIS in the subject cohort achieved favorable functional outcomes (mRS 0–2) on discharge as compared to 51.6% in the control group (*p* = 0.018) (Table 3).

Significantly higher NIHSS scores 24 h after reperfusion therapy (16 (5–24) vs. 5 (2–13), *p =* 0.003) and on day 7 or discharge (15 (5–21) vs. 4 (1–10), *p <* 0.001) were evident in the COVID-19 group as compared to matched controls. The detail outcome data are shown in Table 3. Rate of cerebral edema after the reperfusion treatment did not differ between the two groups, and no sICHs were observed. Both in-hospital and 3 month mortality rates were significantly higher in the COVID-19 group compared to controls (29% and 54.8% vs. 6.5% and 12.9%, *p* = 0.043 and *p* = 0.001, respectively).

The analysis of in-hospital mortality patients in both groups showed severe stroke from onset (baseline NIHSS > 15). COVID-19-positive stroke patients who died in hospital: 5/9 (55.6%) underwent MTE and 4/9 (44.4%) underwent bridging therapy, 2/9 (22.2%) had unsuccessful MTE (TICI 1 and 2a), 7/9 (77.8%) had acute ischemic changes on CT scan 24 h after RT, 2/9 (22.2%) experienced reperfusion complications (small scattered petechiae and subarachnoid hemorrhage, confluent petechiae), 5/9 (55.6%) had various degree cerebral edema, 8/9 (88.9%) had pneumonia and respiratory failure, 2/9 (22.2%) had other somatic complications (sepsis, acute kidney failure and urinary tract infection), 2/2 (100%) control group stroke patients who died in hospital underwent MTE, and reperfusion therapy was successful (TICI 3) in both cases, Both patients had acute ischemic changes on CT scan 24 h after RT, both experienced reperfusion complications (hematoma within infarcted tissue, occupying <30%, intraventricular hemorrhage), both had cerebral edema, and both had pneumonia and respiratory failure and no other somatic complications.

### 3.3. COVID-19 Associated Complications

Severe respiratory failure was observed in 64.5% of COVID-19 patients during any time point of inpatient treatment, and it was significantly more common compared to controls, where only 22% of patients were in respiratory compromise (*p =* 0.007). Importantly, on admission, rates of respiratory failure did not differ between the two groups (hypoxemia rate 5 (16.1%) in COVID-19 group vs. 3 (9.7%) in controls, *p* = 0.712). Pneumonia complicated the disease course of 67.7% of COVID-19 patients as compared to 8% of controls *(p <* 0.001). Prolonged stay in ICU was observed in 38.7% of COVID-19 patients compared to 19.4% in control group (*p* = 0.093).

### 3.4. Multivariate Analysis

The accuracy of a favorable functional outcome prediction was 83.6%. The significant variables in the univariate analysis included age (*p* = 0.028), baseline NIHSS (*p* < 0.001), and COVID-19 infection (*p* = 0.011). In the multivariable model, only baseline NIHSS retained significance (OR 0.790; 95% CI 0.691–0.902) (Table 4).

The accuracy of 3-month mortality after stroke and reperfusion therapy was 78.8%. The significant variables included age (*p* = 0.022), hypoxemia (*p* = 0.079), baseline NIHSS (*p* = 0.001), COVID-19 infection (*p* = 0.001), total WBC count (*p* = 0.079), and CRP concentration (*p* = 0.093). Increasing age and higher baseline NIHSS on admission were associated with a higher likelihood of 3-month mortality after stroke and reperfusion therapy. COVID-19 infection increased the likelihood of death 3 months after stroke and reperfusion therapy seven times (OR 6.696; 95% CI 1.029–43.584), while hypoxemia, total WBC count, and CRP concentration were not significant predictors (Table 5).

## 4. Discussion

This is the first Lithuanian nationwide pair-matched multicenter study evaluating outcomes of COVID-19-positive AIS patients treated with reperfusion therapies. We demonstrated that COVID-19 stroke patients present with a significantly higher neurologic burden than non-infected controls. We also found that reperfusion therapies appear safe for COVID-19 stroke patients in relation to reperfusion-associated complications (symptomatic ICH and cerebral edema). Despite successful reperfusion, the COVID-19 stroke patients had significantly worse outcomes and a high 3-month mortality rate as compared to control patients. We additionally report 3-month mortality of COVID-19-positive patients with AIS representing distant sequalae of AIS. Hypoxia had a major role in our COVID-19 cohort and may have contributed to the high in-hospital and 3-month mortality rate.

Outcomes of COVID-19 patients with AIS seem to be universally unfavorable despite successful reperfusion. Although COVID-19 patients with mild stroke presentations seemed to have more favorable outcomes, in general, COVID-19 patients with AIS were more severely disabled, with a median NIHSS of 15 at discharge as compared to controls. This is in line with other studies reporting in-hospital mortality rates ranging from 31% to 60% [14,15,16]. The European multicenter EVT study provided data on 30-day mortality of 27% [17]. In contrast, we report insights on 3-month mortality even higher than previously reported [18].

In our study, the absolute majority of COVID-19 stroke patients had a more severe stroke despite no differences in ASPECTS scores between study groups on admission. These results are comparable to previous reports [18]. However, the true size of ischemic territory in COVID-19 patients may be larger than initially anticipated. Significantly lower ASPECTS scores and higher infarct volumes were observed for COVID-19 patients with AIS on MRI despite early imaging in a previous study [19]. In contrast, we used CT as our main screening modality. Although discordances between MRI and CT median ASPECTS scores in non-COVID-19 AIS have been documented, no impact to overall outcomes was observed [20]. Therefore, COVID-19-specific endothelial dysfunction may have a role in infarct core size expansion and contribute to poor outcomes.

Moreover, in our study, we demonstrated that COVID-19 stroke patients eligible for reperfusion therapies had prolonged onset-to-door times. Prolonged ODT in COVID-19 patients might be explained by human factors: first, the lack of available paramedical teams on-call could have delayed arrival to the hospital. Second, both stroke admission rates and prolonged ODT were previously reported owing to the reluctance of stroke patients to seek medical care, especially during the start of the pandemic when vaccination was not yet available [21]. However, the impact of prolonged ODT on stroke severity is debatable. Prolonged ODT might also be explained in part by the expanded intervention window for EVT according to the DAWN trial, demonstrating the undeniable benefits of EVT beyond 6 h for rigorously selected patients [22]. However, this approach was not validated for COVID-19 patients, but despite the lack of evidence, the DAWN criteria were applied according to best clinical practice and consensus statements valid at the time of therapy [23,24]. Second, data regarding the efficacy of EVT beyond 6 h in COVID-19 stroke patients are conflicting, since there are no studies specifically addressing this issue in the COVID-19 population. Studies specifically addressing reperfusion beyond 6 h are required to assess their safety and efficacy profile and more importantly, assess the impact of COVID-19 in these patients, especially in cases with respiratory compromise.

In our study, DTN and DTP times did not differ significantly between patients infected with COVID-19 and controls. Every stroke center was pre-notified about COVID-19 positivity in cases when information was available to the paramedical team and when stroke teams made safety preparations in advance. However, in most cases, COVID-19 status was unknown. Treatment of stroke and reperfusion therapy was considered a priority and did not cause delays in logistics in the emergency departments in either of the stroke centers.

Another aspect to consider is early neurological improvement after reperfusion therapy. In our cohort, successful reperfusion (TICI 2b or TICI 3) was observed in 79.2% of COVID-19 patients with AIS who underwent EVT, and in all but one patient (95.5%) in the control group. In addition, the rate of ischemic changes on CT scan 24 h after RT did not differ between COVID-19 and control groups. Despite successful and timely reperfusion, COVID-19 stroke patients did not improve neurologically 24 h after reperfusion. We acknowledge the possibility that some patients may have exhibited a higher neurological burden due to their severe general state and the need for intensive care due to COVID-19. We did not calculate the ICU severity scores to represent the general state of these patients. However, NIHSS scores were evaluated either at 7 days or on discharge for every patient. At these time points, the absolute majority of patients were discharged from the ICU. Therefore, we believe that evaluation of NIHSS later in the disease course more accurately reflects the true neurologic burden. Moreover, a lack of early neurological improvement was observed in other studies owing to several factors. Early consecutive ischemic strokes or re-occlusions of the same vessel after successful or complete recanalization were observed at a higher than expected rate of 8% in a systematic study [25]. In our cohort, we have no data regarding early re-occlusions in COVID-19 stroke patients, since this was a retrospective study and we do not routinely perform CTA after successful reperfusion according to national guidelines, unless there is a high clinical suspicion of re-occlusion.

Another proposed explanation for no neurological improvement is the difference in clot composition in COVID-19 and non-COVID-19 patients. Wang et al. described several patients with excessive clot fragmentation and distal migration during thrombectomy. Moreover, once evaluated with thromboelastography, the thrombi showed features of high clot consolidation and reduced time of clot formation consistent with a severe procoagulant state [26]. Several other studies reported a hypercoagulable state in COVID-19 patients as compared to controls, which may attribute to both the devastating multivessel occlusions, clot fragmentation, consecutive ischemic strokes, or early re-occlusions of blood vessels that might contribute to poor outcomes [27]. Although we cannot confirm the different clot features for COVID-19 stroke patients in our study, other aspects of these patients are worth considering.

Hypoxia is a major contributing factor to poor outcomes in AIS patients. In our cohort, 64.5% of COVID-19 stroke patients suffered from respiratory failure. Almost one-third of COVID-19 patients with AIS required prolonged intubation due to severe respiratory system compromise. In a subgroup analysis of the former group (unpublished data), patients in whom the respiratory function was severely affected were those who showed no neurologic improvement 24 h after reperfusion. Most of these patients presented with LVOs and required EVT for reperfusion. Due to a relatively small sample size in our cohort, we could not perform a subgroup analysis with optimal statistical power, but a tendency toward more severe strokes in patients with severe respiratory compromise was observed. This is in line with previous reports. Two meta-analyses showed that severe COVID-19 disease is more often complicated by severe ischemic strokes [16,28]. It is proposed that patients with severe respiratory compromise can be deemed as high risk for poor outcomes and in-hospital mortality [15]. A stroke center in New York reported good early neurological improvement in COVID-19 stroke patients who underwent endovascular treatment. None of the COVID-19 stroke patients who dramatically improved showed signs of respiratory distress [29]. Respiratory function, although analyzed in AIS with COVID-19 cohorts, has not been widely addressed in the subpopulation of patients undergoing reperfusion therapies for AIS. In our study, we emphasize the importance of respiratory complications for AIS patients undergoing specialized treatment. Respiratory failure could be an important factor for early neurological deterioration or lack of improvement despite successful reperfusion. Novel strategies involving optimal management of respiratory compromise should be exploited to improve the outcomes for stroke patients undergoing reperfusion therapy.

Although available safety evidence is scarce, reperfusion in cases of AIS was recommended by an international panel of experts [23,24]. For IVT, various studies report sICH rates from 2.8% to 10% in COVID-19 stroke patients [30,31,32,33]. As for EVT, a European multicenter retrospective study of 93 COVID-19 stroke patients reported a rate of sICH of 5.4% [17]. In contrast, results from the largest to date EVT trial MR CLEAN reports sICH rates of 7.7%, although differences between the two studies’ sample sizes have to be taken into account [34]. Results from our study are comparable to the aforementioned studies and provide additional insights into the safety of reperfusion therapies for COVID-19 stroke patients. All ICHs were asymptomatic in the COVID-19 group and did not differ statistically from controls. As given the information provided, reperfusion therapies appear to be safe and beneficial for some patients, but large prospective trials evaluating both the safety and efficacy of these treatments are warranted.

Risk factors associated with high dependency and mortality in COVID-19 AIS patients include older age, COVID-19 infection, and stroke severity on admission. The logistic regression model in our study showed only higher baseline NIHSS to be associated with worse functional outcomes. As for 3-month mortality, age, higher baseline NIHSS and COVID-19 infection were significant predictors in the logistic regression model. COVID-19 infection increased the likelihood of death 3 months after stroke and reperfusion therapy seven times. We acknowledge that the regression analysis model in our study may not reflect the true predictors of poor outcomes in COVID-19 AIS patients undergoing RT due to the retrospective nature of the study, data shortages, and a small sample size. Furthermore, we included to our univariate and multivariate logistic regression only patient history data and clinical and laboratory data evaluated on admission. Earlier, we argued that hypoxia is an important factor for the expansion of infarcted brain tissue and may be associated with poor outcomes given the high rates of severe respiratory failure in our study. This might explain the higher rates of in-hospital mortality. However, for the survivors, the causes of 3-month mortality rates remain to be validated.

**Strengths.** The strength of our study lies within a couple of points. First, the study was conducted across all Lithuanian stroke centers. Second, we added valuable insights to the available safety data of reperfusion therapies in AIS with COVID-19 demonstrating relative safety of all treatment modalities. We have performed one of the few studies reporting COVID-19 patients with AIS mortality at 3 months. As a result, it was possible to compare COVID-19 patients with AIS with controls demonstrating clear differences in mortality and functional outcomes, raising COVID-19 as a potential risk factor predicting poor outcomes in AIS patients.

**Limitations.** The major weaknesses of our study are the retrospective nature and a relatively small sample size, restricting subgroup analysis of reperfusion modalities and evaluation of outcomes within. Another weakness is the chosen pair-matched analysis method, which might not accurately represent the true demographic and stroke-specific data of the control patients. We could not perform a subgroup analysis of different treatment modalities that would have added additional safety and outcome data. The regression analysis model, albeit significant for some factors, we believe, does not reflect all predictors of poor outcomes in COVID-19 patients. Heterogeneity between different centers concerning treatment management of patients with AIS should be considered. Although we reported 3-month mortality rates, we could not compare functional outcomes of surviving COVID-19 stroke patients to the control group, which would provide additional information on distant effects of COVID-19 on AIS survivors.

## 5. Conclusions

In conclusion, reperfusion therapies on AIS in COVID-19 patients appear to be safe and should be used. COVID-19-positive AIS patients seem to have more debilitating strokes from onset. Despite successful and timely reperfusion, they tend to have poor functional outcomes with high in-hospital and 3-month mortality rates. For the surviving patients, studies to compare functional outcomes in the post-acute COVID phase between COVID-19 patients with AIS and non-infected stroke survivors are needed.

## Figures and Tables

**Table 1:** Patient demographic data and stroke characteristics.

	Stroke Patients with COVID-19 (*n* = 31)	Control Group without COVID-19 (*n* = 31)	*p* Value
**Female, *n* (%)**	20 (64.5)	20 (64.5)	1.000
**Mean Age, Years (SD)**	74.0 (12.9)	73.7 (12.3)	0.912
**Cardiovascular Risk Factors, *n* (%)**			
Hypertension	29 (93.5)	26 (83.9)	0.425
Dyslipidemia	15 (48.4)	23 (74.2)	0.067
Smoking	5 (16.1)	2 (6.5)	0.229
Diabetes	6 (19.4)	2 (6.5)	0.255
Atrial Fibrillation	12 (38.7)	19 (61.3)	0.075
Symptomatic ICA Stenosis	6 (19.4)	2 (6.5)	0.255
Intracranial Artery Stenosis	3 (9.7)	5 (16.1)	0.707
**Circulation of Stroke, *n* (%)**			
Anterior Circulation	28 (90.3)	28 (90.3)	1.000
Posterior Circulation	3 (9.7)	3 (9.7)	1.000
**Reperfusion Treatment, *n* (%)**			
IVT	7 (22.5)	7 (22.5)	1.000
EVT	15 (48.4)	15 (48.4)	1.000
Bridging Therapy	9 (29.1)	9 (29.1)	1.000
**Median Timeliness Metrics, min (IQR)**			
**Onset-To-Door Time**	126 (83–218)	95 (66–205)	0.294
IVT	94 (81–137)	80 (55–105)	
EVT	245 (121–720)	154.5 (67.75–198.75)	
Bridging Therapy	101 (65–130.5)	84 (67.75–220)	
**Door-To-Needle Time**	40.5 (26–72.5)	36 (27–46)	0.626
**Door-To-Puncture Time**	101 (80.75–162.5)	116.5 (75.5–138.75)	1
**Baseline NIHSS, Median (IQR)**	16 (10–19)	12.5 (5–15)	**0.028**
**ASPECTS, Median (IQR)** ^§^	9 (7.75–10)	10 (8–10)	0.229

SD—standard deviation, ICA—internal carotid artery, IV—Intravenous thrombolysis, EVT—endovascular treatment, mRS—modified Rankin Scale, IQR—interquartile range, NIHSS—National Institutes of Health Stroke Scale, ASPECTS—Alberta Stroke Programme Early CT Score. ^§^ Sample size differs for both subjects (*n* = 30), and control group (*n* = 27) due to missing data. Bold values denote statistical significance at the *p* < 0.05 level.

**Table 2:** Baseline clinical and laboratory data.

	Stroke Patients with COVID-19 (*n* = 31)	Control Group without COVID-19 (*n* = 31)	*p* Value
**Clinical Data**			
Hypoxemia, *n* (%) ^†^	5(16.1)	3 (9.7)	0.712
Median Body Temperature, °C (IQR)	36.6 (36.4–36.8)	36.5 (36.1–36.6)	**0.025**
Mean Systolic Blood Pressure, mmHg (SD)	159 (28.6)	168 (28.6)	0.214
Mean Diastolic Blood Pressure, mmHg (SD)	86 (21.8)	90 (14.9)	0.350
**Laboratory Data**			
Mean Total WBC Count, ×10⁹/L (SD)	8.8 (5.4)	8.7 (2.6)	0.473
Mean Lymphocyte Count, ×10⁹/L (SD)	1.5 (0.7)	2.1 (1.4)	**0.013**
Mean CRP, mg/L (SD)	44.3 (63.8)	5.3 (6.4)	**<0.001**
CRP > 5 mg/L, *n* (%)	23 (74.2)	8 (25.8)	**<0.001**
Median D-Dimer, μg/L (IQR)	675 (78–4898)	1048 (479–2065)	0.979

IQR—interquartile range, SD—standard deviation, WBC—white blood cells, CRP—C-reactive protein. ^†^ Defined as SpO2 < 93%. Bold values denote statistical significance at the *p* < 0.05 level.

**Table 3:** Patient treatment outcomes and complications.

	Stroke Patients with COVID-19 (*n* = 31)	Control Group without COVID-19 (*n* = 31)	*p* Value
**TICI Score, *n* (%) ^†^**			0.190
2b/3	19 (79.2)	21 (95.5)	
0/1/2a	5 (20.8)	1 (4.5)	
**Ischemic Changes on CT Scan 24 h**			
**After RT, *n* (%)**	24 (77.4)	21 (67.7)	0.393
**Stroke Severity, NIHSS, Median (IQR)**			
24 h After Reperfusion Therapy	16 (5–24)	5 (2–13)	**0.003**
24 h Change From Baseline	0 (−3–3)	−2 (−7.25–0)	**0.029**
Day 7 or Discharge ^‡^	15 (5–21)	4 (1–10)	**<0.001**
Overall Change From Baseline	−1 (−6–2)	−4 (−9–1)	**0.022**
**Early Neurological Improvement, *n* (%)** ^§^	6 (19.4)	12 (38.7)	0.077
**Functional Outcome at Discharge ^||^**			
Median mRS (IQR)	4 (3–6)	2 (1–4)	**0.004**
mRS ≤ 2, *n* (%)	7 (22.6)	16 (51.6)	**0.018**
**Complications, *n* (%)**			
Symptomatic ICH	0 (0)	0 (0)	1.000
Cerebral Edema	7 (22.6)	6 (19.4)	0.755
Pneumonia ^¥^	21 (67.7)	2 (8.0)	**<0.001**
Respiratory Failure ^¥¥^	20 (64.5)	4 (22.2)	**0.007**
Other ^¶^	8 (25.8)	9 (29.0)	0.776
**Prolonged Stay in ICU (>1 day), *n* (%)**	12 (38.7)	6 (19.4)	0.093
**Mortality, *n* (%)**			
In-Hospital	9 (29.0)	2 (6.5)	**0.043**
Day 90	17 (54.8)	4 (12.9)	**0.001**

TICI—thrombolysis in cerebral infarction, NIHSS—National Institutes of Health Stroke Scale, IQR—interquartile range, mRS—modified Rankin Scale, ICH—intracerebral hemorrhage, ICU—intensive care unit. ^†^ Only patients who had undergone mechanical thrombectomy (*n* = 46, data of 2 patients was missing). ^‡^ Whichever occurred first. ^§^ Defined as reduction of NIHSS score by 4 points or more or score 0–1 at 24 h after reperfusion therapy. ^||^ Sample size differs for both subjects (*n* = 26) and control group (*n* = 29) due to missing data. ^¥^ Sample size differs for both subjects (*n* = 31) and control group (*n* = 25) due to missing data. ^¥¥^ Sample size differs for both subjects (*n* = 31) and control group (*n* = 18) due to missing data. ^¶^ Including urinary tract infection, pulmonary embolism, myocardial infarction, acute heart failure, pulmonary oedema, other organ dysfunction, major bleeding (excluding pneumonia and respiratory failure). Bold values denote statistical significance at the *p* < 0.05 level.

**Table 4:** Logistic regression model on the likelihood of favorable functional outcome (mRS 0–2) on discharge (*n* = 61).

Covariates	Univariate Analysis	Multivariate Analysis	
	*p* Value	OR (95% CI)	*p* Value
**Age**	0.028	0.959 (0.899–1.022)	0.199
**Baseline NIHSS**	<0.001	0.790 (0.691–0.902)	**0.000**
**COVID-19 Infection**	0.011	0.312 (0.077–1.260)	0.102

OR—odds ratio, CI—confidence interval, NIHSS—National Institutes of Health Stroke Scale. Bold values denote statistical significance at the *p* < 0.05 level in multivariate analysis.

**Table 5:** Logistic regression model on the likelihood of 3-month mortality after stroke and reperfusion therapy (*n* = 52).

Covariates	Univariate Analysis	Multivariate Analysis	
	*p* Value	OR (95% CI)	*p* Value
**Age**	0.022	1.086 (1.002–1.178)	**0.045**
**Hypoxemia (SpO2 < 93%)**	0.079	1.861 (0.225–15.406)	0.565
**Baseline NIHSS**	0.001	1.184 (1.013–1.383)	**0.034**
**COVID-19 infection**	0.001	6.696 (1.029–43.584)	**0.047**
**Total WBC count**	0.079	1.126 (0.829–1.530)	0.447
**CRP concentration**	0.093	1.004 (0.990–1.018)	0.586

OR—odds ratio, CI—confidence interval, NIHSS—National Institutes of Health Stroke Scale, WBC—white blood cells, CRP—C-reactive protein. Bold values denote statistical significance at the *p* < 0.05 level in multivariate analysis.

## Data Availability

Not applicable.
